# Exploring Signs and Symptoms Associated with Meibomian Gland Dysfunction for Use as Clinical Trial Endpoints

**DOI:** 10.1089/jop.2023.0064

**Published:** 2023-11-02

**Authors:** Layla Ajouz, Ashley Nguyen, Cathy Zhao, Michael R. Robinson, Kelly K. Nichols

**Affiliations:** ^1^ Allergan, an AbbVie company, Irvine, California, USA.; ^2^ School of Optometry, University of Alabama at Birmingham, Birmingham, Alabama, USA.

**Keywords:** dry eye disease, endpoints, meibomian gland dysfunction, meibum quality

## Abstract

**Purpose:**

Dry eye disease is attributed to impaired tear production and/or evaporative dry eye. Evaporative dry eye is frequently associated with meibomian gland dysfunction (MGD). The objective of this study was to identify clinical study endpoints related to MGD.

**Methods:**

This 22-day, noninterventional, case–control clinical study involved three cohorts with increasing MGD severity: no MGD, mild/moderate MGD, and severe MGD. Symptoms were assessed with an ocular symptom questionnaire grading blurred vision, eye burning, eye dryness, eye pain, light sensitivity, eye itching, eye foreign body sensation, and overall ocular discomfort. Sign assessments included the maximum meibum quality score (MMQS), tear breakup time, Schirmer tear tests, biomicroscopy, and corneal staining. Signs and symptoms were compared between cohorts and study visits.

**Results:**

Seventy-five study participants were assigned to the cohorts (25 per cohort). MMQS scores increased with increasing MGD severity, reflecting the selection criteria for the cohorts. Between-visit scores showed a weighted kappa statistic of 0.72 indicating substantial agreement. Mean scores of all assessed symptoms increased with increasing MGD severity. Scores for symptoms showed moderate (κ = 0.41–0.60) to substantial (κ = 0.61–0.80) agreement between visits. Overall ocular discomfort demonstrated the strongest correlation with the MMQS.

**Conclusion:**

The MMQS was a reproducible sign of MGD showing good agreement with ocular symptoms. Overall ocular discomfort was well correlated with typical dry eye symptoms and could potentially be used as a single measure of MGD symptoms. The findings from this observational study may inform endpoints for future clinical trials. ClinicalTrials.gov NCT01979887.

Dry eye disease (DED) can be classified as aqueous-deficient dry eye, evaporative dry eye, or both.^1^ Evaporative dry eye is frequently associated with meibomian gland dysfunction (MGD), which has been defined by the International Workshop on Meibomian Gland Dysfunction as “a chronic, diffuse abnormality of the meibomian glands characterized by terminal duct obstruction and/or qualitative/quantitative changes in glandular secretion” that may result in alteration of the tear film, eye irritation, clinically apparent inflammation, and ocular surface disease, including dry eye.^2^ MGD may be primary and unassociated with other local or systemic disease, or it may be secondary to a range of disorders such as acne rosacea, seborrheic dermatitis, psoriasis, and cicatricial conjunctivitis.^3^ Age is an important risk factor for MGD,^4^ and in a large chart review study in Austria, ∼80% of patients who presented with MGD-associated DED were over 40 years of age.^5^

A deficiency in the tear film lipid layer leads to the evaporative tear loss associated with MGD.^6^ Pharmaceutical products addressing this deficiency are in development, but no pharmaceutical products to date have been approved with an indication to treat dry eye associated with MGD.^7,8^

In the United States, the Food and Drug Administration (FDA) issued a draft guidance document in 2020 for industry seeking to gain regulatory approval of drugs in development for treatment of DED.^9^ For drug approval, it was recommended that clinical trials show that the drug improves both objective signs and subjective symptoms of DED, with a statistically significant difference between the drug and vehicle demonstrated for at least one prespecified sign of dry eye (e.g., corneal staining, conjunctival staining, decreased tear breakup time, and decreased Schirmer test score) and at least one prespecified symptom of dry eye (e.g., blurred vision, light sensitivity, sandy or gritty feeling, ocular irritation, ocular pain or discomfort, and ocular itching).^9^ Alternatively, clinical trials could demonstrate a statistically significant difference between the drug and vehicle in the percentage of patients achieving a complete resolution of corneal staining or at least a 10-mm increase in Schirmer test scores.^9^

Given the lack of validated endpoints specifically related to MGD,^10^ primary endpoints related to signs and symptoms of DED (corneal fluorescein staining and ocular dryness, respectively) are being used in phase 3 clinical trials (ClinicalTrials.gov: NCT04139798, NCT04567329, NCT05515471) conducted for regulatory approval of product indications related to treatment of DED associated with MGD.

To guide MGD treatment decisions and the assessment of treatment effectiveness, the Tear Film and Ocular Surface Society (TFOS) International Workshop on Meibomian Gland Dysfunction developed a grading scale for MGD disease severity based on meibum expressibility, quality of the secretions, the presence and severity of symptoms, and corneal fluorescein staining results.^11^ Building upon this work, we conducted a noninterventional clinical study (with two visits after screening activities) exploring signs and symptoms related to MGD to identify outcome measures associated with the presence and severity of MGD that potentially could be used as endpoints in drug registration trials. In addition, we sought to identify correlations between signs and symptoms of MGD.

## Methods

This was a prospective, multicenter (two sites in the United States, one site in England), noninterventional clinical study consisting of two study visits after screening activities in individuals with and without MGD. The study was conducted in accordance with the International Conference for Harmonisation guidelines, applicable regulations, and the Declaration of Helsinki. Institutional Review Board or Ethics Committee approval was obtained at each site before the study was initiated, and all participants provided written informed consent before any study-related assessments. The study is registered at ClinicalTrials.gov with the identifier NCT01979887.

The investigators in the trial were licensed optometrists with PhD degrees and a U.S. board–certified ophthalmologist. All were recognized dry eye experts who had participated in numerous clinical trials in ocular surface disease and had expertise in evaluating all the outcomes measures required in this protocol. In addition, protocol-specific training for all investigators was performed by the sponsor before the study began.

### Participant selection and assignment to cohorts

Eligibility for the study was evaluated at a screening visit and the enrollment (day 1) study visit. Key inclusion criteria included male or female, age 40 years or older before the enrollment visit. Because primary MGD is age-related and typically occurs in patients that are over 40 years of age, we arbitrarily selected this age as the cutoff for study entry. Individuals with uncontrolled ocular disease (except for MGD) or uncontrolled systemic disease were excluded. Other key exclusion criteria, evaluated with respect to the start of the enrollment visit, included history of LipiFlow^®^ or other lid heating therapy, therapeutic gland expression, or meibomian gland probing within the last 12 months; use of a contact lens in either eye within the last 30 days; use of any eyelash growth-stimulating product within the last 30 days; use of systemic or topical macrolides, tetracyclines, or tetracycline-derivative drugs or systemic antihistamines within the last 30 days; and use of any preserved topical artificial tear supplement within the last 30 days or any nonpreserved artificial tear supplement within the last 6 h before the visit.

Individuals who performed lid hygiene within the last 48 h or who wore eye makeup within the last 8 h were also excluded. Individuals who currently used certain systemic vitamins or supplements (those containing omega 3 fatty acids; vitamins A, B, or E; fish oil; or Evening Primrose oil) were excluded if the dosing regimen had not been stable for the last 60 days. A complete listing of all study inclusion and exclusion criteria is provided in Supplementary Table S1.

Screened individuals who had recently used an artificial tear product or prohibited medication, worn eye makeup or contact lenses, or performed lid hygiene were required to undergo a washout interval, and eligibility was reassessed at the enrollment visit scheduled up to 90 days later. For individuals who met all eligibility criteria at the screening visit, screening and enrollment could occur on the same day (day 1).

Individuals who, in the judgment of the investigator, met all eligibility criteria were enrolled in the study, then underwent ocular examinations at the first (enrollment, day 1) study visit. At the end of this visit, the study sponsor reviewed the data collected for selected signs and symptoms. Based on this review, study participants who qualified were assigned to 1 of 3 study cohorts (non-MGD, mild/moderate MGD, and severe MGD) until a sample size of 25 individuals was achieved in each cohort.

The selection criteria used for cohort assignment (Table 1) were consistent with diagnostic criteria and severity grading established by the TFOS International Workshop on Meibomian Gland Dysfunction.^3,11^ These criteria included the maximum meibum quality score (MMQS) obtained in the evaluation of meibum quality of six central meibomian glands in the lower lid, the sum of scores for the worst two symptoms on an ocular symptom questionnaire, and Schirmer test results.

**Table 1.** Cohort Selection Criteria

Cohort	Investigator-graded MMQS^a–c^	Schirmer tear test without anesthesia^b^	Sum of scores of worst 2 symptoms on the ocular symptom questionnaire
Non-MGD	0 or 1	≥7 mm/5 min	0–4 with neither symptom scored as >2
Mild/moderate MGD	2	≥7 mm/5 min	0–4 with neither symptom scored as >2
Severe MGD	3	≥7 mm/5 min	≥4

The cohort selection criteria specified by the latest study protocol amendment are listed.

^a^ Six central glands in the lower lids of each eye were examined and the meibum quality of each gland was graded on a scale of 0 = clear excreta or clear with small particles (normal viscosity); 1 = opaque excreta with normal viscosity; 2 = opaque excreta with increased viscosity (gel-like); 3 = secretions retain shape, or secretions do not completely express but a toothpaste-like substance can be seen at the opening of the orifice; and NE = nonexpressible (nothing at orifice or metaplastic).

^b^ MMQS and Schirmer test criteria must be met in the same eye (the study eye).

^c^ None of the six central glands graded in the eye could have a meibum quality score greater than the maximum specified.

MGD, meibomian gland dysfunction; MMQS, maximum meibum quality score among the six central glands in the lower lid that were graded.

**FIG. 1.**
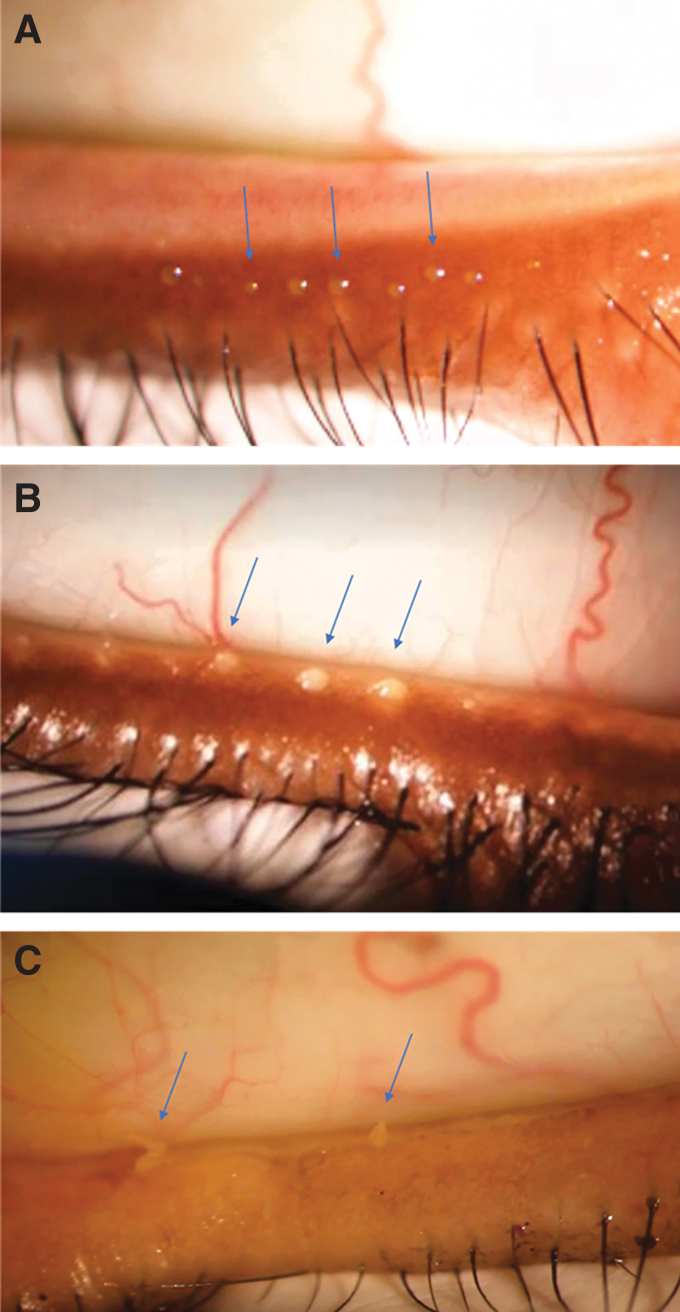
Evaluation of meibum quality in representative study participants. The expressor was placed in the center of the lower eyelid for evaluation of meibum secretion from six glands, and meibum quality was graded on a 4-point scale (0–3). **(A)** Participant with clear excreta of each gland (grade 0). **(B)** Participant with three of the six central glands expressible; the excreta are opaque and have increased, gel-like viscosity (grade 2). **(C)** Participant with two of the six central glands expressible with waxy excreta (grade 3).

To determine the MMQS, the investigator first used a surgical skin marker to physically denote the middle of the lower eyelid. A Meibomian Gland Evaluator (Johnson and Johnson Vision, Irvine, CA, USA) was applied to the base of the central lower eyelid to express meibum from six central meibomian glands of the lower eyelid (Fig. 1), three on each side of the mark placed near/on the lid margin. The investigator then graded the quality of the meibum expressed from each gland on a scale derived from Mathers' meibum quality secretion scale,^12^ with 0 = clear excreta or clear with small particles (normal viscosity); 1 = opaque excreta with normal viscosity; 2 = opaque excreta with increased viscosity (gel-like); 3 = secretions retain shape, or secretions do not completely express but a toothpaste-like substance can be seen at the opening of the orifice; and NE = nonexpressible (nothing at orifice or metaplastic).

MMQS was calculated for each eye as the maximum of the meibum quality scores of the expressible glands among the six central meibomian glands on the lower lid. Meibum quality was normal (MMQS grade 0 or 1) in the non-MGD cohort, poorer (MMQS grade 2) in the mild/moderate MGD cohort, and worst (MMQS grade 3) in the severe MGD cohort. A Schirmer score of at least 7 mm was required in each cohort to exclude individuals with aqueous-deficient dry eye.

To be assigned to a cohort, at least one eye must have met the criteria associated with the cohort, and that eye was designated as the study eye. If both eyes met the criteria for the same cohort, the designation of study eye was based on the sum of the secretion quality scores from expressible glands among the six central meibomian glands in the lower lid. For the non-MGD cohort, the eye with the lower sum of the secretion quality scores was designated as the study eye, and for the mild/moderate and severe MGD cohorts, the eye with the higher sum of the secretion quality scores was designated as the study eye. If the eyes of an individual met the criteria for different cohorts, the eye with the higher MMQS was designated as the study eye, and the individual was assigned to the corresponding cohort if it had not yet met the enrollment goal.

Study participants who were assigned to one of the three cohorts were asked to return to the site for an additional study visit (the exit visit) at day 22 (±3 days). The enrollment and exit visits were scheduled to begin before 2 pm and at the same time of day (within 1 h) to allow enough time to complete the testing before the end of the business day and to reduce the potential for variability in results caused by diurnal variation. Study participants who were not assigned to a cohort (either because they failed to meet the cohort criteria, or because the cohort to which they qualified had already met the enrollment goal of 25 participants) were discontinued from the study after the enrollment visit.

### Exploratory measures

Ocular symptoms were graded by participants at the enrollment and exit visits using a self-administered ocular symptom questionnaire. The questionnaire asked participants to grade the blurred vision, eye burning, eye dryness, eye pain, light sensitivity, eye itching, eye foreign body sensation, and overall ocular discomfort experienced over the past week on a scale of 0–4 with 0 = none, 1 = mild, 2 = moderate, 3 = severe, and 4 = very severe.

Meibum expressibility and the quality of the secretions were evaluated at the enrollment and exit visits as described above. The number of expressible glands in the eye was calculated as the total number of glands (among the six evaluated) with a meibum secretion quality score of 0, 1, 2, or 3.

Other evaluations performed at both the enrollment and exit visits included tear breakup time (TBUT), reading speed measured with the IReST (International Reading Speed Texts) test^13^ using best-corrected near vision refraction, Lissamine Green staining of the upper and lower lid margins, and biomicroscopic evaluation of the upper and lower lids. TBUT was measured at the slit lamp after application of 1 μL of 2% preservative-free liquid NaFl to the lower cul-de-sac of the right eye. The participant was instructed to blink three times, then stare without blinking. Using a stopwatch, the investigator measured TBUT as the time from the last blink until one or more black (dry) spots appeared in the precorneal tear film. The procedure was repeated twice, and a second and third measurement for the right eye was taken. After a 60-s rest period, the entire procedure was repeated for the left eye.

Lissamine Green staining of the upper and lower lid margins was performed for both eyes. For each eye, a Lissamine Green strip was moistened with 0.9% saline, then the strip was gently touched against the superior bulbar conjunctiva of the eye. Staining on the upper and lower eyelid margins was graded 2 min later using the slit lamp at 16 × magnification and a grading scale of 0 = none, 1 = broken line, 2 = thin line, 3 = thick line, and 4 = patch.

Both eyes were examined with biomicroscopy. Biomicroscopic findings of erythema, edema, telangiectasias, notching/irregularity, and other pathology (such as abnormal lid position/closure) were graded on a scale of 0 = none, +0.5 = trace, +1 = mild, +2 = moderate, and +3 = severe. The total (composite) score for gradings of eyelid/eyelid margins/lashes findings (maximum of 15) was calculated separately for the upper and lower lid of each eye.

Tear secretion (measured with Schirmer tests without anesthesia) and corneal sodium fluorescein staining were evaluated in both eyes at the enrollment visit only. For corneal staining, a fluorescein strip was moistened with 0.9% saline, then the strip was gently touched against the superior bulbar conjunctiva of the eye. Staining of the entire cornea was visualized 2 min later at the slit lamp using 10 × magnification, a yellow barrier filter, and a cobalt blue filter for illumination. Corneal staining was graded using the Oxford scheme.^14^

Other exploratory measures (e.g., patient-reported health outcome measures, and assessment of potential biomarkers using spectroscopic analysis of meibum samples) will be reported separately. Safety evaluations included adverse events, vital signs, and best-corrected visual acuity.

### Statistical methods

Analyses used the population of all participants assigned to a study cohort and were based on the cohort assignments. Analysis of Schirmer test scores, TBUT, and reading speed at a study visit used analysis of variance models with cohort and site as factors for pairwise comparisons between cohorts. Paired *t*-tests were used for within-group comparisons between visits. Analysis of MMQS, number of expressible glands, ocular symptom scores, biomicroscopic gradings, Lissamine Green staining scores, and corneal fluorescein staining scores at a study visit used the Cochran–Mantel–Haenszel method with modified ridit scores, stratified by site, for pairwise comparisons between cohorts. Wilcoxon signed-rank tests were used for within-group comparisons between visits.

Relationships between exploratory measures were evaluated using Spearman rank-order correlations. Unweighted and weighted kappa statistics were used to assess the agreement of measurements obtained at the day 1 and day 22 study visits. Kappa values were interpreted as suggested by Landis and Koch, with κ values of ≤0, 0.01–0.20, 0.21–0.40, 0.41–0.60, 0.61–0.80, 0.81–0.99, and 1.00 indicating no, slight, fair, moderate, substantial, near perfect, and perfect agreement, respectively.^15^ As this was an exploratory trial, all statistical comparisons were made at the α = 0.05 level without adjustment for multiple comparisons, and nominal *P* values are provided. Also, because this was an exploratory study, the sample size was determined empirically. A sample size of 25 participants in each cohort (75 total) was planned.

## Results

Among a total of 177 individuals who were screened for the study, 129 met the eligibility criteria and were enrolled into the study across 3 clinical sites. After the enrollment (day 1) study visit, based on day 1 ocular symptom, MMQS, and Schirmer test scores, 75 participants who qualified for a cohort were placed into the non-MGD, mild/moderate MGD, or severe MGD cohort (25 participants per cohort) and were asked to return for a second study visit on day 22. Participants who were not assigned to a cohort (*n* = 54), generally because of failure to qualify for a cohort or because the appropriate cohort already included 25 participants, were discontinued from the study. All but two of the participants assigned to a cohort completed the study as planned; one participant in the mild/moderate MGD cohort discontinued because of a nonocular adverse event, and one participant in the severe MGD cohort, who reported severe dryness and overall ocular discomfort, was lost to follow-up.

Table 2 shows the demographics of the participants in each cohort. Overall, the mean (standard deviation [SD]) age of the participants assigned to cohorts was 54.5 (9.49) years. Two-thirds of these participants were women (66.7% [50/75]), and most were either Black (44.0% [33/75]) or White (30.7% [23/75]); the remainder were Hispanic (14.7% [11/75]), Asian (5.3% [4/75]), or other (5.3% [4/75]).

**Table 2.** Demographics of the Cohorts

Characteristic	Cohort			Total (*n* = 75)
	Non-MGD (*n* = 25)	Mild/moderate MGD (*n* = 25)	Severe MGD (*n* = 25)	
Age, mean (SD), y	52.0 (8.34)	52.8 (6.26)	58.8 (11.86)	54.5 (9.49)
Range	40–74	43–63	41–89	40–89
< 45, *n* (%)	4 (16)	4 (16)	3 (12)	11 (14.7)
45–65, *n* (%)	19 (76)	21 (84)	17 (68)	57 (76)
> 65, *n* (%)	2 (8)	0	5 (20)	7 (9.3)
Sex, *n* (%)				
Male	9 (36)	9 (36)	7 (28)	25 (33.3)
Female	16 (64)	16 (64)	18 (72)	50 (66.7)
Race/ethnicity, *n* (%)				
White	13 (52)	6 (24)	4 (16)	23 (30.7)
Black	7 (28)	15 (60)	11 (44)	33 (44)
Asian	1 (4)	1 (4)	2 (8)	4 (5.3)
Hispanic	1 (4)	2 (8)	8 (32)	11 (14.7)
Other	3 (12)	1 (4)	0	4 (5.3)

SD, standard deviation.

MMQS scores increased with increasing MGD severity (Table 3), reflecting the selection criteria for the cohorts. For each pairwise comparison of cohorts, the difference in the distribution of MMQS scores was statistically significant (*P* < 0.001) at day 22 (exit visit) as well as day 1 (enrollment visit). Mean MMQS scores were comparable at the enrollment and exit visits. Agreement in the MMQS score between visits (i.e., the percentage of participants with the same score at both visits) was 69.9%. The corresponding weighted kappa statistic was 0.72 (95% confidence interval [CI]: 0.62–0.83) indicating substantial (κ = 0.61–0.80) agreement.

**Table 3.** Maximum Meibum Quality Score in the Study Eye

MMQS	Enrollment/day 1			Exit/day 22		
	Non-MGD (*n* = 25)	Mild/moderate MGD (*n* = 25)	Severe MGD (*n* = 25)	Non-MGD (*n* = 25)	Mild/moderate MGD (*n* = 24)	Severe MGD (*n* = 24)
0, *n* (%)	17 (68)	0	0	16 (64)	1 (4.2)	0
1, *n* (%)	8 (32)	0	0	6 (24)	4 (16.7)	0
2, *n* (%)	0	25 (100)	0	2 (8)	16 (66.7)	7 (29.2)
3, *n* (%)	0	0	25 (100)	1 (4)	3 (12.5)	17 (70.8)
*P* value versus non-MGD^a^		<0.001	<0.001		<0.001	<0.001
*P* value versus mild/moderate MGD^a^			<0.001			<0.001
Mean (SD)	0.3 (0.48)	2.0 (0.00)	3.0 (0.00)	0.5 (0.82)	1.9 (0.68)	2.7 (0.46)^b^

Six central glands in the lower lid were graded to determine the MMQS.

^a^ Pairwise comparisons of cohorts were based on the Cochran–Mantel–Haenszel method with modified ridit scores, stratified by site.

^b^ *P* < 0.05 for change from day 1 to 22, based on the Wilcoxon signed-rank test.

Across cohorts, at both the enrollment and exit visits, the number of expressible glands among the six central glands evaluated decreased with increasing severity of MGD (Table 4). On day 1, all 6 glands were expressible in 92% (23/25) of participants in the non-MGD cohort, 64% (16/25) of participants in the mild/moderate MGD cohort, and 44% (11/25) of participants in the severe MGD cohort. On day 22, the percentage of participants with all 6 glands expressible was 88% (22/25) in the non-MGD cohort, 62.5% (15/24) in the mild/moderate MGD cohort, and 29.2% (7/24) in the severe MGD cohort.

**Table 4.** Number of Expressible Glands Among Six Central Glands of the Study Eye

Number of expressible glands	Enrollment/day 1			Exit/day 22		
	Non-MGD (*n* = 25)	Mild/moderate MGD (*n* = 25)	Severe MGD (*n* = 25)	Non-MGD (*n* = 25)	Mild/moderate MGD (*n* = 24)	Severe MGD (*n* = 24)
1, *n* (%)	0	0	1 (4)	0	1 (4.2)	0
2, *n* (%)	0	0	4 (16)	0	0	2 (8.3)
3, *n* (%)	0	1 (4)	5 (20)	0	1 (4.2)	6 (25.0)
4, *n* (%)	1 (4)	3 (12)	1 (4)	2 (8)	4 (16.7)	8 (33.3)
5, *n* (%)	1 (4)	5 (20)	3 (12)	1 (4)	3 (12.5)	1 (4.2)
6, *n* (%)	23 (92)	16 (64)	11 (44)	22 (88)	15 (62.5)	7 (29.2)
*P* value versus non-MGD^a^		0.019	<0.001		0.037	<0.001
*P* value versus mild/moderate MGD^a^			0.032			0.007
Mean (SD)	5.9 (0.44)	5.4 (0.87)	4.4 (1.75)	5.8 (0.58)	5.2 (1.28)	4.2 (1.35)

^a^ Pairwise comparisons of cohorts were based on the Cochran–Mantel–Haenszel method with modified ridit scores, stratified by site.

Results of the evaluation of other signs potentially associated with the presence and severity of MGD are shown in Table 5. Schirmer test scores in the cohorts showed no relationship between tear production and the presence and severity of MGD. Mean corneal fluorescein staining scores followed the trend non-MGD < mild/moderate MGD < severe MGD, but the differences between groups were small and not statistically or clinically significant. Mean composite scores of biomicroscopic eyelid findings for the upper and lower lid were <1 in all cohorts, and there were no consistent differences among the cohorts in individual or overall biomicroscopy findings. Lissamine Green staining scores for the upper and lower lid margins and results of reading speed tests were also similar among the cohorts. Mean TBUT scores followed the trend non-MGD > mild/moderate MGD > severe MGD at day 22, when TBUT scores were significantly lower in the severe MGD cohort compared with the non-MGD cohort (*P* = 0.045), but not at day 1.

**Table 5.** Assessments of Other Signs Potentially Associated with MGD

Exploratory outcome measure, mean (SD)	Enrollment/day 1			Exit/day 22		
	Non-MGD (*n* = 25)	Mild/moderate MGD (*n* = 25)	Severe MGD (*n* = 25)	Non-MGD (*n* = 25)	Mild/moderate MGD (*n* = 24)	Severe MGD (*n* = 24)
Biomicroscopic upper lid findings composite score	0.76 (1.128)	0.34 (0.515)	0.64 (0.919)	0.92 (1.087)	0.31 (0.412)	0.67 (0.868)
Biomicroscopic lower lid findings composite score	0.64 (0.848)	0.22 (0.384)^a^	0.52 (0.669)	0.68 (0.900)	0.35 (0.429)	0.50 (0.590)
Lissamine Green upper lid margin staining score	2.8 (0.83)	2.4 (0.91)	2.8 (0.62)	3.0 (0.35)	2.7 (0.92)	3.0 (0.62)
Lissamine Green lower lid margin staining score	2.8 (0.82)	2.6 (0.76)	2.4 (0.71)	2.7 (0.54)	2.8 (0.88)	2.7 (0.91)
TBUT, s	4.977 (3.7201)	5.605 (3.9994)	4.221 (1.6985)	4.945 (2.6529)	4.228 (1.7895)^b^	4.055 (1.4790)^c^
Reading speed on IReST, words per min	166.12 (30.952)	165.69 (27.580)	147.67 (32.805)	162.27 (32.242)	171.92 (29.281)	148.05 (41.816)
Schirmer tear test score without anesthesia, mm	16.4 (9.36)	18.6 (11.11)	16.7 (9.35)	ND	ND	ND
Corneal fluorescein staining score	1.6 (0.96)	1.9 (1.05)	2.0 (1.04)	ND	ND	ND

^a^ *P* < 0.05 versus non-MGD, based on Cochran–Mantel–Haenszel method with modified ridit scores, stratified by site.

^b^ *P* < 0.05 for change from day 1 to 22, based on paired *t-*test.

^c^ *P* < 0.05 vs non-MGD, based on analysis of variance model with cohort and site as factors.

IReST, International Reading Speed Texts; ND, not done; TBUT, tear breakup time.

All symptoms evaluated on the ocular symptom questionnaire increased with increasing MGD severity (Table 6). Comparisons of the distribution of scores for the seven individual ocular symptoms as well as overall ocular discomfort showed significantly (*P* < 0.05) different distributions in the severe MGD cohort compared with the non-MGD cohort, the mild/moderate MGD cohort, or both, at both days 1 and 22, apart from light sensitivity at day 22. Most scores of the seven individual ocular symptoms and overall ocular discomfort were 0, 1, or 2; grade 3 (severe) symptoms were reported only by participants in the mild/moderate and severe MGD cohorts.

**Table 6.** Mean (Standard Deviation) Scores on the Ocular Symptom Questionnaire

Survey Item^a^	Enrollment/day 1			Exit/day 22		
	Non-MGD (*n* = 25)	Mild/moderate MGD (*n* = 25)	Severe MGD (*n* = 25)	Non-MGD (*n* = 25)	Mild/moderate MGD (*n* = 24)	Severe MGD (*n* = 24)
Blurred vision	0.7 (0.75)	1.1 (0.73)	1.7 (0.54)^b,c^	0.6 (0.65)	1.2 (0.82)^b^	1.6 (0.83)^b^
Burning	0.5 (0.65)	0.7 (0.80)	1.4 (0.87)^c^	0.6 (0.77)	0.8 (0.74)	1.4 (0.93)^c^
Dryness	1.0 (0.82)	1.4 (0.76)	2.0 (0.65)^b,c^	0.9 (0.67)	1.4 (0.88)^b^	1.9 (0.74)^b,c^
Foreign body sensation	0.6 (0.82)	0.9 (0.81)	1.6 (0.99)^b,c^	0.4 (0.58)	0.9 (0.80)	1.6 (0.93)^b,c^
Itching	0.7 (0.74)	0.8 (0.66)	1.8 (0.87)^b,c^	0.7 (0.79)	1.0 (0.86)	1.6 (0.71)^b,c^
Light sensitivity	0.8 (0.78)	1.0 (0.73)	1.8 (0.66)^b,c^	0.8 (0.76)	1.4 (0.83)^b,d^	1.5 (0.66)^d^
Pain	0.4 (0.71)	0.4 (0.57)	1.4 (0.99)^c^	0.3 (0.54)	0.5 (0.66)	1.3 (0.94)^b,c^
Overall ocular discomfort	0.6 (0.65)	1.0 (0.82)	2.0 (0.73)^b,c^	0.6 (0.65)	1.1 (0.88)	1.6 (0.58)^b^
Total score	5.2 (4.52)	7.2 (4.23)	13.7 (4.37)^b,c^	4.8 (4.00)	8.1 (5.46)	12.4 (4.00)^b,c^

^a^ Seven individual symptoms and overall ocular discomfort were graded on a scale from 0 = none to 4 = very severe. Total score is the sum of scores for all eight items (maximum score of 32).

^b^ *P* < 0.05 versus non-MGD, based on Cochran–Mantel–Haenszel method with modified ridit scores, stratified by site.

^c^ *P* < 0.05 versus mild/moderate MGD, based on Cochran–Mantel–Haenszel method with modified ridit scores, stratified by site.

^d^ *P* < 0.05 for change from day 1 to 22, based on Wilcoxon signed-rank test.

The symptom total score, a measure derived from the ocular symptom questionnaire that was calculated by summing the scores for all seven individual symptoms and overall ocular discomfort, also increased with increasing MGD severity (Table 6). At both day 1 and day 22, the distribution of total scores was significantly different between the severe MGD cohort and the non-MGD and mild/moderate MGD cohorts (*P* < 0.05). Mean total score followed the trend severe MGD > mild/moderate MGD > non-MGD.

Within each cohort, symptom scores were similar between day 1 and 22, except for light sensitivity, which increased in the mild/moderate MGD cohort and decreased in the severe MGD cohort (*P* < 0.05). Agreement in scores between visits ranged from 57.5% (for itching) to 72.6% (for burning); agreement in scores was 71.2% for overall ocular discomfort (Supplementary Table S2). The weighted kappa statistic ranged from 0.49 (95% CI: 0.33–0.65) for blurred vision to 0.68 (95% CI: 0.55–0.81) for burning, indicating moderate (κ = 0.41–0.60) to substantial (κ = 0.61–0.80) agreement between visits. The weighted kappa statistic for overall ocular discomfort scores was 0.67 (95% CI: 0.54–0.80).

**FIG. 2.**
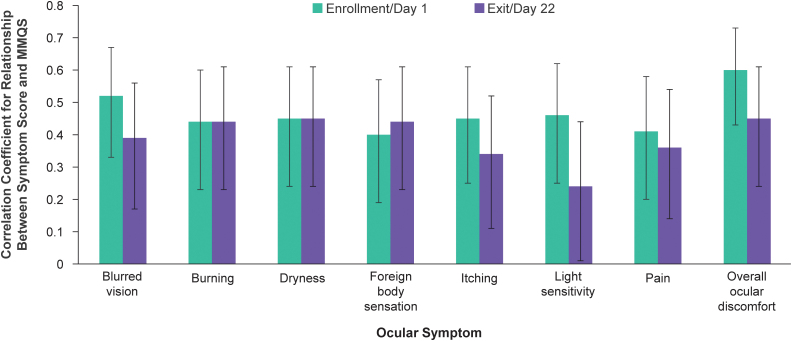
Spearman correlation coefficients for the relationship between ocular symptom scores and the MMQS at each study visit. Errors bars indicate the 95% confidence interval. MMQS, maximum meibum quality score among the six central glands graded.

The strength of the relationship between two variables can be evaluated with correlation analysis. Although the level of correlation considered to be a “strong” or “weak” relationship is subjective and differs between studies,^16,17^ a Spearman correlation coefficient with absolute value of 0.70 may be considered to indicate a strong relationship, while a coefficient with absolute value of 0.40–0.69, 0.10–0.39, and 0.00–0.10 may be considered to indicate a moderate, weak, and negligible relationship, respectively.^17^ In analysis of the correlation between symptom scores and the MMQS, the overall ocular discomfort score and scores of all individual ocular symptoms showed a significant positive correlation with the MMQS at both study visits (Fig. 2). The Spearman correlation coefficients at day 1 and 22, respectively, were 0.52 and 0.39 for blurred vision, 0.44 and 0.44 for burning, 0.45 and 0.45 for dryness, 0.41 and 0.36 for pain, 0.46 and 0.24 for light sensitivity, 0.45 and 0.34 for itching, 0.40 and 0.44 for foreign body sensation, and 0.60 and 0.45 for overall ocular discomfort.

The overall ocular discomfort score also showed a significant but weaker relationship to TBUT at day 22 (Spearman correlation of −0.24, 95% CI: −0.45 to −0.01). Evaluation of relationships between objective signs showed a significant correlation of TBUT with the corneal staining score at day 1 (Spearman correlation of −0.47, 95% CI: −0.63 to −0.27). The only notable association of MMQS with other signs was a significant negative correlation between the MMQS and reading speed at day 1 (Spearman correlation coefficient of −0.31, 95% CI: −0.50 to −0.08).

No study participant experienced a serious or study procedure-related adverse event during the study.

## Discussion

This study explored signs and symptoms that could potentially identify the presence and severity of MGD. The 2007 and 2017 TFOS International Dry Eye WorkShop (DEWS and DEWS II) reports acknowledged that when evaluating an individual with ocular surface complaints, no single sign is sufficiently sensitive and specific to allow diagnosis of DED.^14,18,19^ The assessment of symptoms as well as signs is important, because dry eye is a symptomatic disease, and symptom questionnaires are among the most repeatable of the commonly used diagnostic tests.^18^ Blurred vision, burning, dryness, pain, light sensitivity, itching, and foreign body sensation are symptoms commonly reported by patients with DED,^20^ and, along with overall ocular discomfort, were assessed with the symptoms questionnaire used in our study. In contrast to the U.S. FDA guidance on endpoints for dry eye clinical trials, which suggests the use of endpoints for both a sign and a symptom, or a sign only,^9^ in the European Union, regulators will accept a single symptom endpoint for product approval in DED.^21^

For clinical trials of dry eye associated with MGD, a sign directly related to meibomian gland function may be an ideal endpoint. Both meibum expressibility and the quality of the expressed meibum are altered in MGD.^3^ These changes are related to disease severity and are associated with clinical features of MGD, including decreased tear film stability and ocular surface damage.^3,22^ Importantly, a study by Korb and Blackie^23^ demonstrated a significant negative correlation between a measure of meibum secretion quality (i.e., the number of meibomian glands yielding liquid secretion, MGYLS) and dry eye symptoms.^23^ Meibomian gland assessments have been used as primary endpoints in both drug and device trials in MGD.^24,25^

In this study, the number of expressible glands decreased across cohorts with increasing severity of MGD. There was also a trend across cohorts for increased corneal staining scores associated with the presence and severity of MGD, but the differences in corneal staining scores were not significant. Total corneal fluorescein staining recently was used successfully as a primary endpoint in a clinical trial in patients with DED associated with MGD.^7^ However, data suggest that corneal staining is not a sensitive measure of dry eye,^26^ and many clinical trials in DED have failed to meet endpoints related to corneal staining.^27^

Results of biomicroscopic examinations of the eyelids and Schirmer tests showed no relationship to the presence and severity of MGD. Although morphologic lid signs can be associated with MGD,^22^ mean biomicroscopy composite scores of findings in the upper and lower lids were low in all cohorts. An explanation for this observation may be that individuals with clinically significant signs of eyelid inflammation were excluded from the study, contributing to low composite scores of biomicroscopic lid findings across the three cohorts. The mean Schirmer test scores across cohorts in the study were >16 mm, clearly in the normal range, suggesting that the ocular symptomatology in the MGD cohorts most likely was related to evaporative dry eye.

There were no consistent differences in TBUT across cohorts, which was somewhat surprising, since evaporative dry eye and tear film lipid layer abnormalities in MGD are associated with low TBUT.^3^ These results potentially may be explained by variability in TBUT assessments in a clinical setting. At day 1, TBUT scores were highest in the mild/moderate MGD cohort, but scores in this cohort were decreased significantly at day 22, when TBUT showed the expected trend of non-MGD > mild/moderate MGD > severe MGD. Advanced imaging techniques may allow more reliable assessment of TBUT and better correlation of TBUT with the severity of MGD.^28^

The quality of meibum from each of the six central glands in the lower lid was graded using a scale based on a previously reported grading scale,^12^ and the maximum quality score from expressible glands (MMQS) was analyzed. The MMQS at the day 1 visit increased with MGD severity, reflecting the selection criteria for the cohorts. Because of day-to-day variability in dry eye signs and symptoms in individuals with DED, the study included 2 visits (i.e., day 1 enrollment, and day 22 exit) for assessment of the degree of concordance between measurements at the visits. The MMQS at the day 22 visit also increased with MGD severity. Moreover, the corresponding weighted kappa statistic demonstrated substantial agreement of the MMQS between visits, suggesting that the MMQS is a reliable measure, and supporting its use as a potential sign endpoint in clinical trials of DED associated with MGD.

Infrared meibography potentially could also be used in meibomian gland assessments, as it enables direct observation of meibomian gland morphology and glandular dropout. However, in a study in patients with obstructive MGD, meibum quality, but not gland loss, was significantly related to TBUT and the corneal staining score,^29^ suggesting that a measure of meibum quality may be a better endpoint than gland loss for trials in patients with DED related to MGD.

All symptoms evaluated with the questionnaire used in the study showed higher scores with increasing severity of MGD at both visits. This correlation might be expected because of the use of symptom scores as well as the MMQS in the selection criteria for the non-MGD, mild/moderate MGD, and severe MGD cohorts. However, the goal of this study was to examine the MMQS in “cleanly defined” groups, consistent with the diagnostic criteria and severity grading established by the TFOS International Workshop on MGD. Further studies are indicated to explore the association between the MMQS and DED symptoms in a broader MGD population that does not meet the stringent diagnostic criteria used in this study.

Independent of how participants were categorized into the three cohorts, each individual symptom evaluated with the questionnaire showed a positive correlation with the MMQS at both visits. Importantly, the symptom with the strongest correlation with the MMQS was overall ocular discomfort. The overall ocular discomfort score also showed good test–retest reliability. As overall ocular discomfort was highly correlated with typical DED symptoms evaluated on the questionnaire, it could be used as a single, comprehensive, and representative measure of ocular symptoms in MGD. This would lessen the response burden as individuals could be asked a single question to measure their symptoms associated with MGD.

The lack of a good correlation between the signs and symptoms of DED has frequently resulted in the failure of clinical trials in DED to meet coprimary (sign and symptom) endpoints.^21^ Because it can be difficult to achieve both sign and symptom coprimary endpoints in a trial, the FDA guidance for developing drugs to treat DED states that efficacy for a sign and efficacy for a symptom do not have to be demonstrated in the same clinical trial, but each should be demonstrated in more than one clinical trial.^9^ This results in some sponsors having to conduct at least four registrational trials to meet the regulatory requirements for drug registration.^30^ The correlation of MMQS (sign) and overall ocular discomfort (symptom) demonstrated in our study may simplify the development pathway by enabling the use of MMQS and overall ocular discomfort as coprimary endpoints for an “MGD associated with evaporative dry eye” label. This may potentially reduce the need for conducting more than two registration studies.

To date, regulatory agencies have not accepted composite endpoints consisting of a sign, a symptom, and potentially a biomarker for registration purposes for a dry eye product, so we do not attempt to propose one here. Nevertheless, other therapeutic areas use composite endpoints in drug registration trials for the FDA,^31–34^ and this is an area of potential exploration with regulatory agencies for an “MGD associated with evaporative dry eye” label.

A potential limitation of this study was that participants were assigned to the cohorts based only on their MMQS, Schirmer test scores, and worst scores on the ocular symptom questionnaire, and these signs and symptoms may not encompass the constellation of signs and symptoms that determine MGD severity in all patients with MGD. Measures such as tear osmolarity and tear biomarkers, which potentially could be useful in determining the presence and severity of MGD-related DED,^35–37^ were not included in the study because of practical considerations. Furthermore, many of the study enrollees did not qualify for a cohort assignment, and the relationship between the MMQS and dry eye symptoms in patients with MGD who did not qualify for a cohort was not evaluated. Nevertheless, the study objective was met by the identification of a single sign and a single symptom that could be used as primary endpoints in registrational clinical trials.

Enrollment criteria are used to select patients with a homogeneous disease status. In our study, participants were required to be at least 40 years of age and healthy, except for having MGD. Therefore, the results are applicable to patients at least 40 years of age with MGD and may not be applicable to patients under the age of 40 years and those with MGD occurring secondary to other disorders.

Another study limitation was the small sample size and number of investigational sites. In addition, in this exploratory trial, all statistical comparisons were made at the α = 0.05 level without adjustment for multiple comparisons, and nominal *P* values are reported. Also, studies conducted in Asian populations have shown an apparently higher prevalence of DED compared with studies conducted in the United States,^27,38^ and the applicability of the study results to individuals of Asian ethnicity is not clear, as most of the participants in this study were White or Black, and only four were Asian. Apparent differences among the cohorts at enrollment with respect to race/ethnicity most likely occurred by chance, but could possibly reflect risk factors for MGD and MGD severity. Finally, this study was not designed to evaluate changes in the signs and symptoms of MGD during treatment for MGD-related DED. However, the results suggest that MMQS and overall ocular discomfort may be useful endpoints in future studies of potential treatments for MGD-related DED.

## Conclusions

The MMQS showed good correlation with symptoms in a very defined group of symptomatic MGD patients with abnormal secretions but not abnormal tear production. From the viewpoint of identifying single signs and symptoms that could serve as endpoints in clinical trials and potentially support product registration with a labeled indication for MGD associated with evaporative dry eye, the MMQS was identified as a readily assessable sign related to the pathology and severity of MGD, with high reproducibility between visits and substantial agreement with a variety of DED symptoms. In addition, overall ocular discomfort correlated with typical DED symptoms and could be used as a single comprehensive measure of the symptoms of MGD. The usefulness of MMQS and overall ocular discomfort as coprimary endpoints for clinical trials in MGD-related DED should be confirmed in a larger study involving a diverse population of patients with MGD.

## Supplementary Material

Supplemental data

Supplemental data

## Investigators and Sites

Kelly K. Nicholls and Daniel Powell (Houston, TX, USA); Michel Guillon (London, United Kingdom); Kenneth Sall (Artesia, CA, USA).

## Data Sharing Statement

AbbVie is committed to responsible data sharing regarding the clinical trials we sponsor. This includes access to anonymized, individual, and trial-level data (analysis data sets), as well as other information (e.g., protocols, clinical study reports, or analysis plans), as long as the trials are not part of an ongoing or planned regulatory submission. This includes requests for clinical trial data for unlicensed products and indications.

These clinical trial data can be requested by any qualified researchers who engage in rigorous, independent, scientific research, and will be provided following review and approval of a research proposal, Statistical Analysis Plan (SAP), and execution of a Data Sharing Agreement (DSA). Data requests can be submitted at any time after approval in the United States and Europe and after acceptance of this article for publication. The data will be accessible for 12 months, with possible extensions considered. For more information on the process or to submit a request, visit the following link: https://www.abbvieclinicaltrials.com/hcp/data-sharing/
