# Supplementary material for: Exploring Signs and Symptoms Associated with Meibomian Gland Dysfunction for Use as Clinical Trial Endpoints
Source: J Ocul Pharmacol Ther. 2023 Nov 2;39(9):611–21. doi: 10.1089/jop.2023.0064 (PMC10654652; doi:10.1089/jop.2023.0064)
Supplement: Supplemental data [file Suppl_TableS1.docx]

**Supplementary Table S1. Eligibility Criteria for All Study Participants**

| **Inclusion Criteria** |
| --- |
| 1. Written informed consent has been obtained prior to any study-related assessments. |
| 1. Written documentation has been obtained in accordance with the relevant country and local privacy requirements, where applicable (eg, Written Authorization for Use and Release of Health and Research Study Information [US] and data protection [EU]) prior to any assessments. |
| 1. Male or female, 40 years of age or older prior to the enrollment visit (day 1). |
| 1. In the opinion of the investigator, subject has the ability to follow study instructions and is able to complete all study assessment tools (eg, Subject Ocular Symptom Questionnaire, Meibomian Gland Dysfunction Symptoms Questionnaire, Meibomian Gland Dysfunction Impact Questionnaire) without any assistance or alteration to the assessment tools. |
| 1. In the opinion of the investigator, subject has the ability to complete all required procedures planned during the study. |
| **Exclusion Criteria** |
| 1. Uncontrolled ocular disease (except for MGD) or uncontrolled systemic disease. |
| 1. The presence of any significant ocular or systemic autoimmune illness or condition that could, in the judgment of the investigator, jeopardize subject safety or interfere with interpretation of the study results (eg, Sjögren’s syndrome and related autoimmune diseases). |
| 1. Female who is pregnant or nursing, or planning a pregnancy during the study. |
| 1. Known hypersensitivity to any components of the procedural treatments, including sodium fluorescein and lissamine green. |
| 1. Current enrollment in an investigational drug, device, or observational study or participation in such a study in the 30 days prior to the screening visit (present study excluded). |
| 1. Current active ocular infection or non-keratoconjunctivitis sicca (KCS) ocular surface inflammation (eg, episcleritis), or intraocular inflammation (eg, iritis) in either eye in the 90 days prior to the screening visit. |
| 1. KCS secondary to the destruction of conjunctival goblet cells (as with vitamin A deficiency or scarring) in either eye (such as that with cicatricial pemphigoid, limbal stem cell deficiency, graft versus host disease, alkali burns, Stevens-Johnson syndrome, trachoma, or irradiation). |
| 1. Other corneal disorder or abnormalities in either eye other than those caused by MGD, including those that may markedly affect corneal sensitivity or normal spreading of the tear film (eg, corneal transplant, epithelial basement membrane dystrophy). |
| 1. Subjects with 1 or 2 prosthetic eyes. |
| 1. Eyelid abnormalities (eg, ptosis, previous chalazion incision, palsy, eyelid malposition, abnormal lid closure) in either eye other than those caused by MGD. |
| 1. History of herpes keratitis (simplex or zoster) in either eye. |
| 1. Subjects who have undergone any of the following procedures within the designated time frame relative to the enrollment visit (day 1) or anticipate the need for such procedures during the study: 2. Punctal plugs removed in either eye within 60 days prior to the enrollment visit (day 1) or anticipated removal during any portion of the study. 3. Punctal plugs present within 60 days prior to the enrollment visit (day 1) or anticipated insertion during any portion of the study. 4. Subjects with a history of punctal cautery performed in either eye at any time prior to the enrollment visit (day 1) or who anticipate such a procedure during the study. 5. LipiFlow or other lid-heating therapy, meibomian gland probing, or therapeutic gland expression in either eye within 12 months of the enrollment visit (day 1). 6. History of anterior segment surgery or trauma that could affect corneal sensitivity (eg, cataract surgery or any surgery involving a limbal or corneal incision) in either eye within the last 12 months prior to the enrollment visit (day 1). 7. Use of contact lens in either eye within 30 days prior to the enrollment visit (day 1), or anticipated contact lens wear in either eye during the study. 8. History of corneal refractive surgery within the last 2 years prior to the enrollment visit (day 1) in either eye, or persistent ocular discomfort (eg, ocular dryness symptoms associated with refractive surgery) in either eye following refractive surgery that, in the opinion of the investigator, is considered to be related to the surgery. 9. Subjects who had permanent upper and/or lower eyelid tattooing on either eye at any time prior to the enrollment visit (day 1). 10. Subjects who had oculoplastic surgery on either eye or eyelid at any time prior to the enrollment visit (day 1). |
| 1. Subjects with clinically significant anterior blepharitis, meibomitis or ocular rosacea in either eye that required local or systemic drug treatment (including use of antibiotics, retinoids and/or anti-inflammatory agents) in the 30 days prior to the enrollment visit (day 1), or who anticipate the need for such therapies during the study. |
| 1. Subjects who have performed lid hygiene within 48 hours prior to the enrollment visit (day 1). |
| 1. Subjects who wear eye makeup within 8 hours prior to the enrollment visit (day 1). |
| 1. Subjects who use LATISSE or other eyelash growth-stimulating product within 30 days prior to the enrollment visit (day 1) or anticipate use during the study. |
| 1. Subjects who use systemic or topical macrolides, tetracyclines or tetracycline derivative drugs (including doxycycline and minocycline) within 30 days prior to the enrollment visit (day 1) or anticipate use during the study. |
| 1. Subjects who currently use or have used topical ocular medications within 30 days prior to the enrollment visit (day 1) or anticipate use during the study. |
| 1. Subjects who currently use or have used any preserved topical artificial tear supplement (eg, solutions, gels, ointments) within 30 days prior to the enrollment visit (day 1) or anticipate use during the study. |
| 1. Subjects who used any nonpreserved artificial tear supplement (eg, solutions, gels, ointments) within 6 hours prior to the enrollment visit (day 1). |
| 1. Subjects who currently use or have used systemic antihistamines within 30 days prior to the enrollment visit (day 1) or anticipate use during the study. |
| 1. Subjects who currently use or have used calcineurin inhibitors (eg, ocular cyclosporine) within 30 days prior to the enrollment visit (day 1) or anticipate use during the study. |
| 1. Subjects who currently use or have used systemic 5-alpha reductase inhibitors within 90 days prior to the enrollment visit (day 1) or anticipate use during the study. |
| 1. Subjects who currently use or have used systemic androgen replacement therapy within 90 days prior to the enrollment visit (day 1) or anticipate use during the study. |
| 1. Subjects who currently use or have used systemic inhibitors of steroidogenesis, spironolactone, cyproterone acetate, or other anti-androgen treatment within 90 days prior to the enrollment visit (day 1) or anticipate use during the study. |
| 1. Subjects who are currently on the following systemic medications, vitamins or supplements of which a stable dosing regimen is not established (dosing is not stable if a subject starts, stops, or changes dose and/or drug within the timeframe as described below, or if a subject anticipates starting, ending or changing a regimen during the study)    1. Within 30 days prior to the enrollment visit (day 1)       1. Antimuscarinics, diuretics, antidepressants, or cholesterol lowering agents.    2. Within 60 days prior to the enrollment visit (day 1)       1. Systemic beta blocking agents or cholinergic agonists.       2. Systemic vitamins and/or systemic supplements containing omega 3 fatty acids; vitamins A, B, and E; fish oil; or Evening Primrose oil.    3. Within 90 days prior to the enrollment visit (day 1)       1. Hormone-replacement therapy program, oral or transdermal contraception use, use of other estrogen or progesterone treatment. |
| 1. Subjects who have a condition or are in a situation that, in the investigator's opinion, may put the subject at significant risk, may confound the study results, or may interfere significantly with the subject's participation in the study. |
