# Supplementary material for: Exploring Signs and Symptoms Associated with Meibomian Gland Dysfunction for Use as Clinical Trial Endpoints
Source: J Ocul Pharmacol Ther. 2023 Nov 2;39(9):611–21. doi: 10.1089/jop.2023.0064 (PMC10654652; doi:10.1089/jop.2023.0064)
Supplement: Supplemental data [file Suppl_TableS2.docx]

**Supplementary Table S2. Agreement in Ocular Symptom Scores Between the Enrollment (Day 1) and Exit (Day 22) Visits**

| **Ocular Symptom** | **Percentage Agreement^a^** | **Weighted Kappa (95% CI)** |
| --- | --- | --- |
| Blurred vision | 60.3% (44/73) | 0.49 (0.33, 0.65) |
| Burning | 72.6% (53/73) | 0.68 (0.55, 0.81) |
| Dryness | 60.3% (44/73) | 0.52 (0.37, 0.67) |
| Foreign body sensation | 63.0% (46/73) | 0.58 (0.44, 0.71) |
| Itching | 57.5% (42/73) | 0.50 (0.36, 0.65) |
| Light sensitivity | 63.0% (46/73) | 0.54 (0.38, 0.69) |
| Pain | 67.1% (49/73) | 0.56 (0.40, 0.73) |
| Overall ocular discomfort | 71.2% (52/73) | 0.67 (0.54, 0.80) |

^a^Proportion of participants with the same score at both visits.

CI, confidence interval.
